# Mild Form of Treacher Collins Syndrome Imitating Juvenile Otosclerosis

**DOI:** 10.1155/2012/616797

**Published:** 2012-08-16

**Authors:** Karol Zeleník, Pavel Komínek

**Affiliations:** ^1^Department of Otorhinolaryngology, University Hospital Ostrava, 708 52 Ostrava, Czech Republic; ^2^Faculty of Medicine, University of Ostrava, 703 00 Ostrava, Czech Republic

## Abstract

Treacher Collins syndrome (TCS) is an inherited developmental disorder. More than 40% of individuals with TCS have conductive hearing loss attributed to external and middle ear anomalies. Mild cases of TCS often pass undiagnosed at birth or early childhood. The disease may be manifested as conductive hearing loss in teenagers and may resemble juvenile otosclerosis. Patients could suffer from slight facial variabilities including retrognathia (as in our case) and others, which point out to a possible middle ear anomaly. Surgical corrections of middle ear anomalies including TCS generally lead to poorer outcomes comparing with juvenile otosclerosis, which should be discussed with parents during preoperative counselling.

## 1. Introduction

Treacher Collins syndrome (TCS) is an inherited autosomal dominant disorder with a prevalence estimated at 1 in 40000–70000 of live births. About 60% of patients have the disorder as the result of a de novo gene mutation. In the vast majority of cases, full expressivity of the syndrome occurs, and TCS is clearly diagnosed at birth. However, patients with mild form of TCS may remain undiagnosed for a long time [1].

## 2. Case Report

**Figure 1:**
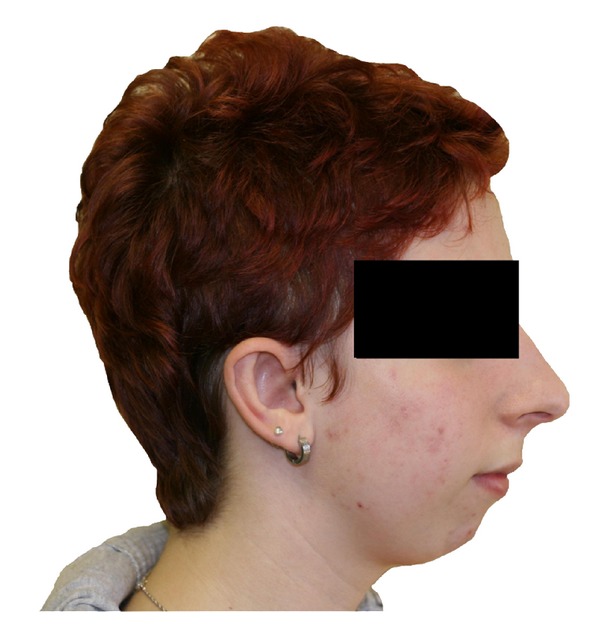
Mild form of Treacher Collins syndrome (TCS). Patient did not present other facial abnormalities except mild retrognathia.

**Figure 2:**
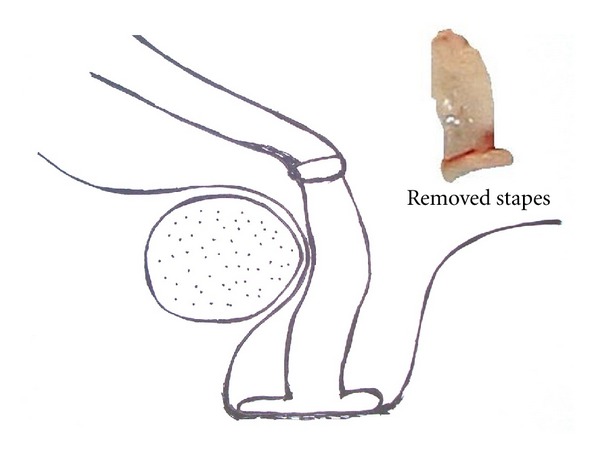
Schema of the right middle ear: stapes ankylosis, monopodial stapes with small footplate and facial canal displaced anteriorly. Removed stapes in the upper right-hand side of the picture.

**Figure 3:**
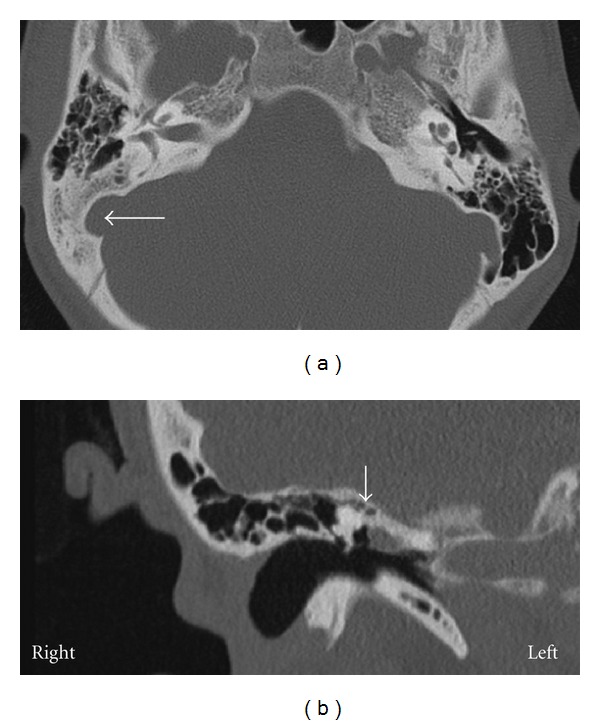
Computed tomography of the temporal bone. (a) Coronal section: lowered pneumatization of the mastoid (long horizontal arrow). (b) frontal section: narrow epitympanum with fixation of the ossicular chain (short vertical arrow).

A 15-year-old female patient was presented with a 3-year long right side hearing loss without dizziness or tinnitus. There was neither history of any trauma, previous ear surgery, or ear infection, nor was there any abnormality of the pinna, ear canal, or the tympanic membrane. However, mild retrognathia was noticed (Figure 1). A pure tone audiogram during the first consultation showed a moderate conductive right side hearing loss with mean air conduction value of 50 dB and mean air-bone gap 35 dB at frequencies 0, 5, 1, 2, and 3 kHz. Left ear audiogram was normal. Tympanometry showed type A bilateral curve with no stapedial reflexes. Audiometry at 6 months later showed slight deterioration (5 dB) of the hearing level in the right ear. Juvenile otosclerosis was considered, and explorative tympanotomy was performed. During the surgery, stapes ankylosis, monopodial stapes with a small footplate, fixed incus, and facial canal displaced anteriorly were revealed. The facial canal was pressed on monopodial stapes, and there was no dehiscence, adhesion, or bony bar (Figure 2). Monopodial stapes were removed, and total ossicular replacement prosthesis (TORP) was used for the reconstruction (placed on fascia tissue). High-resolution computer tomography (HRCT) of the temporal bones was done postoperatively, and multiple pathologies of the middle ear were revealed (Figure 3). Genetic testing confirmed diagnosis of TCS. Because family history was negative, we can assume that de novo gene mutation occurred in this case. Audiogram at 1 year after the surgery showed mean air conduction value of 23 dB and mean air-bone gap 8 dB. Patient is now 3 years after surgery, and hearing is stable. 

## 3. Discussion

Middle ear abnormalities occur in approximately 20%–25% as a part of a malformation syndrome of which TCS is one of the most frequently diagnosed [2, 3]. TCS is most often characterized by hypoplasia of the zygomatic bones and the mandible, external ear abnormalities, coloboma and absence of the cilia of the lower eyelid, and preauricular hair displacement [1]. Other less common abnormalities include cleft palate with or without cleft lip and unilateral or bilateral choanal stenosis or atresia. More than 40% of individuals with TCS have conductive hearing loss attributed to external and middle ear anomalies.

Cremers and Teunissen noticed that specific syndromes are associated with characteristic middle ear malformations [2, 4]. They report on 2 patients with TCS having ankylotic stapes pressed on the bony wall of the facial nerve that was detached and mobilized successfully [2, 4]. We observed a similar pathology in our patient. 

The most common HRCT findings in patients with TCS are narrow epitympanum, abnormalities of the ossicular chain, abnormal course of the facial nerve, and absence of mastoid pneumatization, which is a rather constant finding (2/3 of all ears) [1]. All of the above mentioned findings were present in our patient (Figure 3).

It might be challenging to distinguish between juvenile otosclerosis and congenital ossicular problem (as those attributed to TCS) during preoperative examination [3]. There are some indices, which can help but also mislead the physician, including family history of surgery for hearing loss and the age of manifestation which are not very reliable indicators [3, 5].

Very important information for distinguishing between juvenile otosclerosis and minor congenital middle ear malformation is progression of the hearing loss. This is only detectible when the child undergoes regular examinations and cooperates well. Progressive hearing loss is typical for juvenile stapes otosclerosis, while nonprogressive hearing loss is typical for middle ear malformations [3]. However, audiogram is subjective and might be sometimes confusing, as we can see from our case report.

Other very useful information, which should point out to possible middle ear malformation, is the presence of even slight facial variability (in our case retrognathia). That is why even minor facial variabilities with conductive hearing loss should lead us to think of a middle ear anomaly and indicate HRCT as an important part of preoperative assessment. Suspicion of congenital middle ear anomaly is particularly important for giving appropriate information (generally poorer hearing outcomes of the surgery) to the parents during preoperative counseling, and hearing aid should be offered as an alternative option [3, 5]. However, the final diagnosis can be made only during the surgery.

The technique of middle ear surgical reconstruction for combined middle ear anomalies depends on the level of the middle ear findings and in comparison with stapedotomy for juvenile otosclerosis generally leads to poorer outcomes, comparable to middle ear reconstruction for chronic ear disease [3, 5]. In our patient TORP placed on fascia was used with excellent result, that is why we recommend this technique to be used for reconstruction in patients with ankylotic monopodial stapes, fixed incus, and displacement of the facial bony canal, commonly seen in patients with TCS.
